# Treatment of Infected Pseudoaneurysm of Femoral Artery after Vascular Closure Device Deployment: A Practical Solution

**DOI:** 10.1155/2012/292945

**Published:** 2012-10-18

**Authors:** Predrag Matic, Srdjan Babic, Slobodan Tanaskovic, Dario Jocic, Djordje Radak

**Affiliations:** Dedinje Cardiovascular Institute, Medical School, University of Belgrade, 11000 Belgrade, Serbia

## Abstract

Like other invasive procedures, percutaneous coronary interventions are associated with complications. Most common access site for these procedures is common femoral artery. Complications such as groin and retroperitoneal hematoma can be encountered as well as pseudoaneurysms, arteriovenous fistulas, acute arterial occlusion, and infection. When infected pseudoaneurysm occurs, surgical treatment can be extremely difficult. We present a case of the patient in whom infected pseudoaneurysm of common femoral artery developed after percutaneous coronary intervention and was successfully treated by surgical excision and autoarterial graft insertion.

## 1. Introduction 

Like other invasive procedures, percutaneous coronary interventions are associated with complications. According to the literature data, complication rates related to access artery puncture are different, mostly because of lack of standardized criteria for establishing the diagnosis [1]. Most common site for access is common femoral artery, followed by radial and at the end brachial artery which is rarely used [2]. Incidence of complications associated with femoral artery puncture is estimated around 2–6% [2]. As complications, groin and retroperitoneal hematomas can be encountered as well as pseudoaneurysms, arteriovenous fistulas, acute arterial occlusion, and infection [2]. Development of infection at puncture site can be potentiated by more frequent use of vascular closure devices such as AngioSeal [2]. We present a case of the patient in whom infected pseudoaneurysm of common femoral artery developed after percutaneous coronary intervention and was successfully treated by surgical excision and autoarterial graft insertion. 

## 2. Case Report 

**Figure 1:**
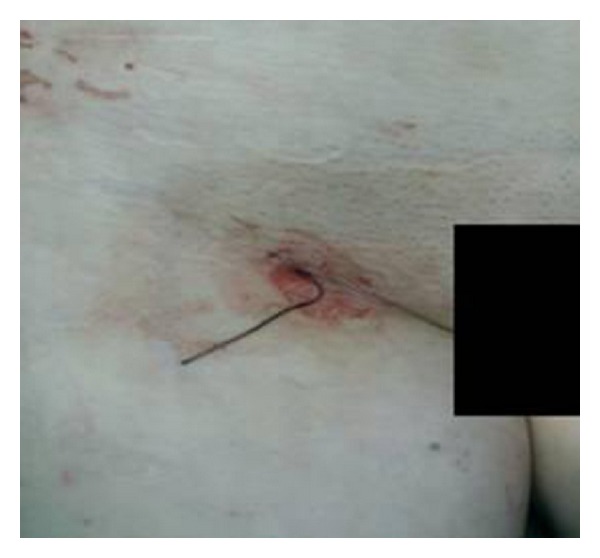
Pseudoaneurysm with puss discharge.

A 63-years-old female patient was admitted at our Institute due to evaluation of stable angina pectoris. Her past medical history included arterial hypertension, hyperlipidemia, and heavy smoking. After performing clinical examination, echocardiography, and coronarography, indication for angioplasty of ramus interventricularis anterior was established. Percutaneous coronary intervention (PCI) was succesfully performed, and two coronary stents were deployed during the procedure. Arterial access was obtained through right common femoral artery, and at the end of intervention, vascular closure device (AngioSeal) was deployed. The next day patient was discharged from the Institution in good condition. Three weeks after the discharge, the patient was readmitted due to dehydratation, poor general condition, and fever (38°C). Clinical examination revealed presence of pulsating mass in the right groin of 3 cm in diameter, and punctiform wound in center with puss discharge (Figure 1). 

**Figure 2:**
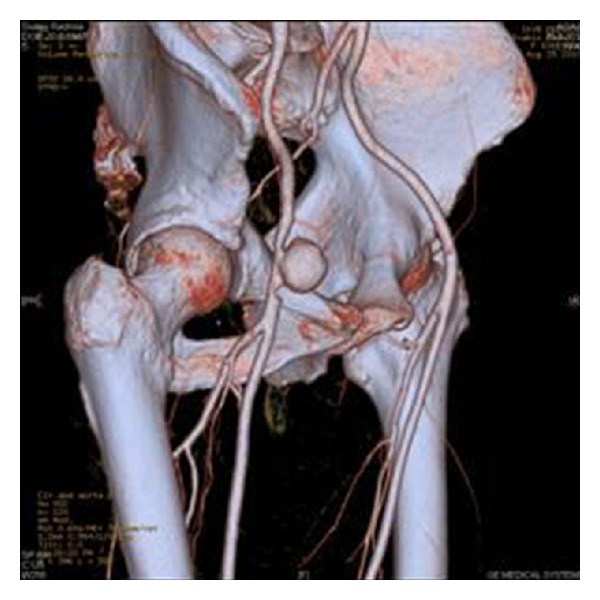
Pseudoaneurysm of right common femoral artery on CT angiography.

Laboratory results showed leucocytosis (16 × 109/L) and elevation of C-reactive protein to 130 mg/l. Hemocultures that were obtained were negative. Ultrasonography and CT angiography verified presence of pseudoaneurysm of right common femoral artery (2.5 cm in diameter) (Figure 2). 

**Figure 3:**
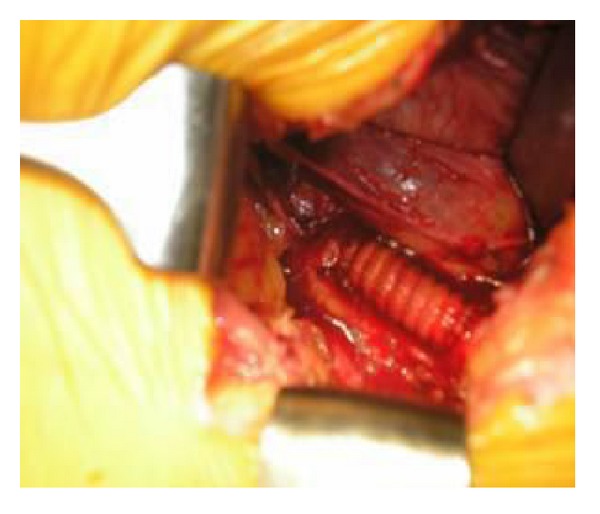
Reconstruction of external iliac artery with silver graft.

After short preoperative preparation, the patient underwent surgical intervention under general anaesthesia. Double sterile preparation of operative field was performed. Oblique incision above inguinal ligament was used to access, extraperitoneally, external iliac artery. Artery was dissected about 5 cm in length. Intravenous heparin (5000 IU) was administered. After clamping, 3 cm of external iliac artery was resected. The defect was reconstructed by interposition of tubular silver graft (diameter 7 mm) (Figure 3). 

**Figure 4:**
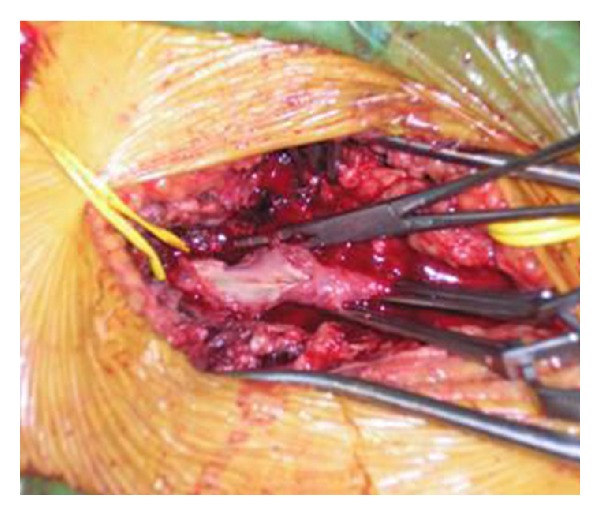
Destruction of anterior wall of common femoral artery due to infection process.

The wound was then closed and protected with gauze. Longitudinal incision in the right groin is then performed to access femoral arteries. Common femoral, profunda femoris, and superficial femoral artery were dissected as well as pseudoaneurysm. After clamping and resection of pseudoaneurysm total destruction of anterior wall due to infection process of common femoral artery in length of about 2 cm was noted (Figure 4).

**Figure 5:**
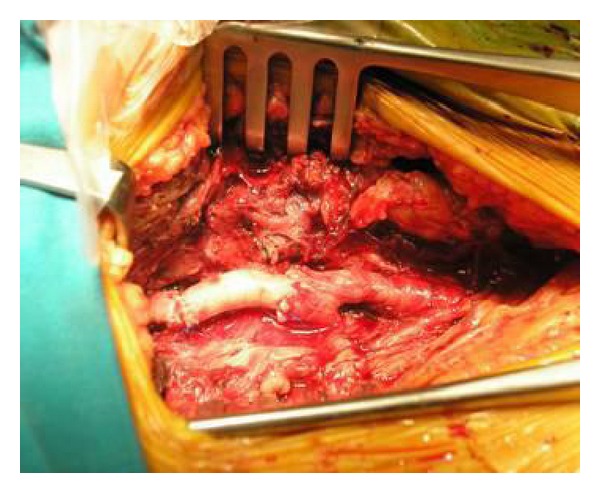
Reconstruction of common femoral artery by autoarterial graft interposition (previously prepared iliac artery).

Reconstruction was made by autoarterial graft interposition (previously prepared iliac artery) (Figure 5).

**Figure 6:**
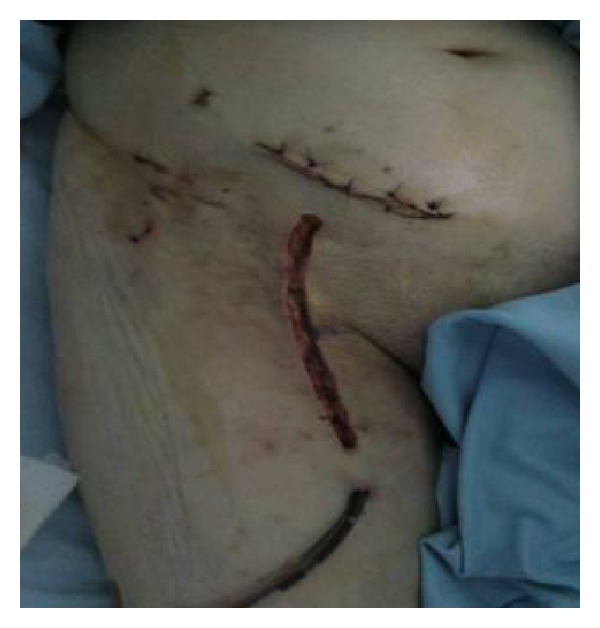
Wounds at the end of surgery.

The wound was reconstructed in layers without closing the skin (Figure 6). 

**Figure 7:**
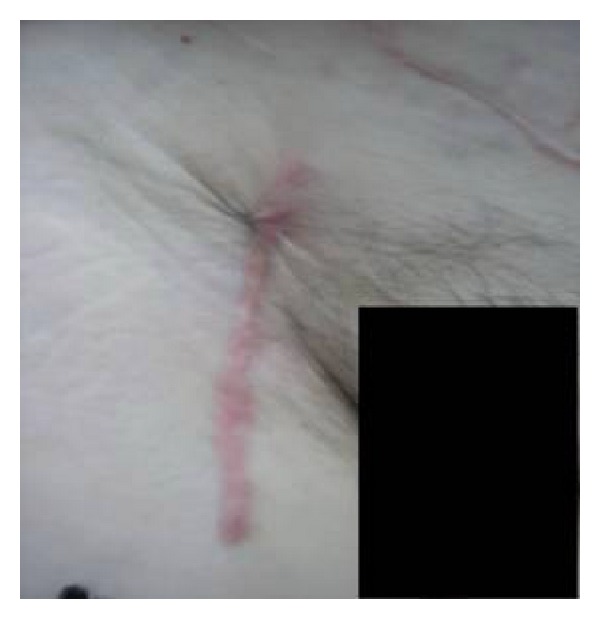
Healed wound during followup.

Further postoperative course was uneventful with normalization of laboratory markers of inflammation. Antibiotics were administered according to the results of, intraoperatively obtained, wound swab (*Staphylococcus aureus* isolated). On the seventh postoperative day, groin skin was sutured, and few days after, the patient was discharged. During six months follow-up period, patient was doing well with healed wounds (Figure 7) and pedobrachial index 1.0. 

## 3. Discussion 

Although recently published, meta-analyses showed no superiority of vascular closure devices over manual compresion [3, 4], their use has dramatically risen in the last years in order to reduce incidence of access site complications, patient discomfort, and time of immobilization [5]. AngioSeal is consisted of anchor made of absorptive polymer and trombin clot which is put to arterial surface using suture. Important complications, such as infection in groin, occlusion of femoral artery, hematoma, and pseudoaneurysm, associated with its use develop in about 2% of patients [6–8]. Those complications occur due to learning curve of its use or device malfunction. With femoral artery punction, pseudoaneurysms can develop in up to 7.5% of cases and can cause distal embolization, external compression on neurovascular structures, rupture, or hemorrhage [9]. Smaller hematomas are common and usually do not need treatment. If the hematoma is larger, ultrasonography can reveal presence of pseudoaneurysm. It can be treated by compression with or without ultrasound guidance. If it persists even after compression, surgery is indicated [10]. Recently published meta-analysis [11] showed increased risk of complications when vascular closure devices, such as AngioSeal, are used. Presence of infection, additionally, makes surgical treatment difficult. Geary et al. [12], as well as Pipkin et al. [13], report several types of *Staphylococcus* that were isolated from wound swabs and hemocultures. Although blood cultures in our case were negative, they can be positive in up to 86% of cases [14]. Sprouse et al. describe cases of infection of vein patch in patient that was treated by extraanatomic bypass surgery [15]. In those conditions, the use of synthetic grafts in not desirable, which makes these reconstructions hard and nonstandard [16]. In this short report, we described one of possible practical solutions in dealing with infected groin pseudoaneurysms as a consequence of PCI and use od AngioSeal as vascular closure device. Of course, when such complication occurs, treatment must be established individually for each patient.
